# Differential Involvement of the Dentate Gyrus in Adaptive Forgetting in the Rat

**DOI:** 10.1371/journal.pone.0142065

**Published:** 2015-11-03

**Authors:** Mickaël Antoine Joseph, Nicolas Fraize, Jennifer Ansoud-Lerouge, Emilie Sapin, Christelle Peyron, Sébastien Arthaud, Paul-Antoine Libourel, Régis Parmentier, Paul Antoine Salin, Gaël Malleret

**Affiliations:** 1 University Lyon 1, University Lyon 1, Lyon, France; 2 Centre National de la Recherche Scientifique (CNRS), Unité Mixte de Recherche 5292, Lyon, France; 3 Institut National de la Santé et de la Recherche Médicale (INSERM), Unité 1028, Lyon, France; 4 Lyon Neuroscience Research Center (CRNL), Lyon, France; Nathan Kline Institute and New York University School of Medicine, UNITED STATES

## Abstract

How does the brain discriminate essential information aimed to be stored permanently from information required only temporarily, and that needs to be cleared away for not saturating our precious memory space? Reference Memory (RM) refers to the long-term storage of invariable information whereas Working Memory (WM) depends on the short-term storage of trial-unique information. Previous work has revealed that WM tasks are very sensitive to proactive interference. In order to prevent such interference, irrelevant old memories must be forgotten to give new ones the opportunity to be stabilized. However, unlike memory, physiological processes underlying this adaptive form of forgetting are still poorly understood. Here, we precisely ask what specific brain structure(s) could be responsible for such process to occur. To answer this question, we trained rats in a radial maze using three paradigms, a RM task and two WM tasks involving or not the processing of interference but strictly identical in terms of locomotion or motivation. We showed that an inhibition of the expression of *Zif268* and *c-Fos*, two indirect markers of neuronal activity and synaptic plasticity, was observed in the dentate gyrus of the dorsal hippocampus when processing such interfering previously stored information. Conversely, we showed that inactivating the dentate gyrus impairs both RM and WM, but improves the processing of interference. Altogether, these results strongly suggest for the first time that the dentate gyrus could be a key structure involved in adaptive forgetting.

## Introduction

For many years, scientists have been investigating the neural bases of memory. A cardinal distinction lies between long-term/Reference Memory, and short-term/Working Memory. Reference Memory (RM) refers to the long-term storage of invariable information gradually acquired over many training sessions whereas Working Memory (WM) depends on the short-term storage of trial-unique information [1–4]. The mechanisms underlying these forms of memory have often been studied separately; some authors have studied the neural bases of WM while others have tried to determine the biological correlates of the long-term storage of information [5, 6]. However, a key question remains: how does the brain distinguish information important enough to be consolidated into long-term memory from information required only temporarily, and that needs to be cleared away for not saturating our cognitive resources?

Some authors have suggested that WM would be more a form of forgetting than a form of memory [7]. Working memory would thus be a short-term memory that, once used, should be forgotten or ignored. In fact, forgetting is intrinsic to the nature of WM [8]: as information is supposedly only temporarily stored in WM, this means that, after a while, such information is not available any longer. We recently proposed that WM and RM could simply be two antagonistic processes, one requiring forgetting and the other impaired by it [9]. Using a transgenic mouse model, we showed that forebrain expression of an inhibitor of the protein phosphatase 2A (PP2A) constrains hippocampal long-term synaptic transmission (LTD) and forgetting [10]. Inhibiting PP2A thus blocked the expression of LTD and the abilities of the mice to forget old information (concerning a platform position learned in the water maze task) that was no longer relevant. This deficit of forgetting also impaired WM abilities in a T-maze task by increasing the level of interference between trials. On the contrary, we showed that hippocampal expression of an inhibitor of the cAMP-dependent protein kinase (PKA) limits hippocampal long-term synaptic transmission (LTP) and RM, but also increases LTD, forgetting and WM abilities by decreasing the level of interference between highly identical trials of a radial maze task [9]. These results thus suggest that the long-term storage of information into RM could benefit from phosphorylation mechanisms increasing LTP, while forgetting and the processing of interference would depend on dephosphorylation and LTD.

**Fig 1:**
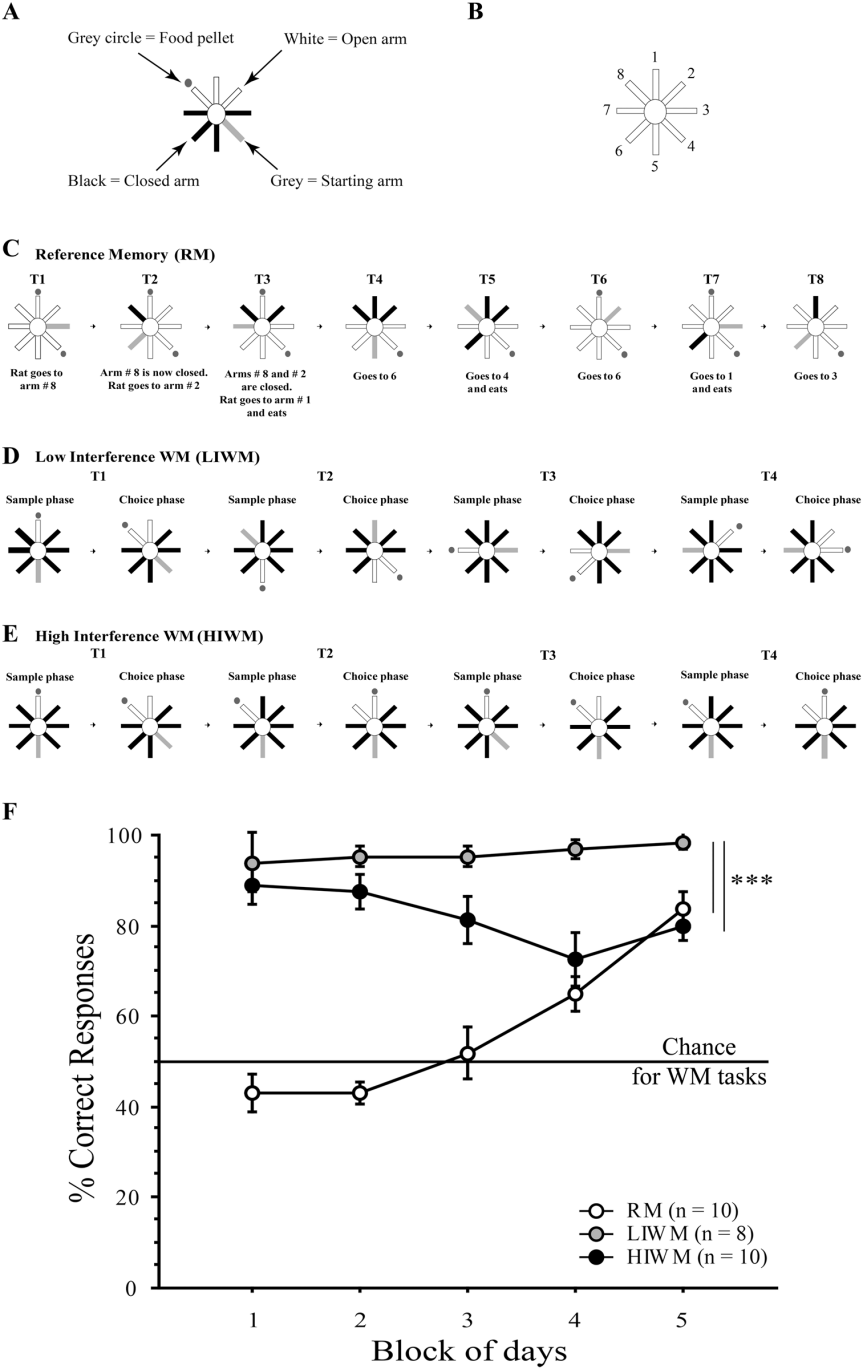
The build-up of proactive interference impairs the performance of rats trained in the HIWM task over time. (A-B) schematic representations of the maze with the arms’ attributed numbers and legends. (C-E) Behavioral paradigms (see Material and Methods) and schematic representation of one training day for each of the experimental groups. (C) Reference memory (RM) training. The same two arms (here 1 and 4, (B)) are baited every day for each trial. Each daily session consisted of 8 trials (T1 to T8). (D) Low Interference Working Memory (LIWM) training. Each day consisted of 4 trials (T) of 2 phases each. (E) High Interference Working Memory (HIWM) training. The same pair of arms is used every day for each trial. Consequently, the trials are very similar to each other and it is therefore necessary to ignore previous trials in order to complete an ongoing trial. (F) Behavioral performances of each group of rats. Percentage of correct choices ± s.e.m per Block (each Block = 2 days of training) in RM, LIWM and HIWM tasks. Black line represents the chance level for both WM tasks.

During the past decades, numerous studies have considerably advanced our understanding of memory processes and their cellular and molecular underpinnings [11, 12]. The concept of forgetting, however, remains elusive, probably because forgetting has often been seen as just a lack of memory, a failed process that happens to us involuntarily [8, 13, 14]. Human studies suggest just the opposite and propose that forgetting is as important as memory, and that some forms of forgetting are adaptive and essential to secure optimal storage of information [7, 14–19]. For instance, forgetting previous orders for a waiter taking many similar orders during a shift seems essential for the storage of relevant (e.g. new orders) information. Such examples of the adaptive role of forgetting are numerous. Forgetting has often been studied in humans using directed forgetting, a paradigm explicitly asking the subject to forget information [20, 21]. However, animal models are essential to decipher the cellular or molecular underpinnings of forgetting. As we cannot explicitly ask an animal to forget, our goal was to find a way to determine such bases of adaptive forgetting, in particular in the context of WM processing. To do so, and instead of studying this process in an isolated way, we adopted a comparative approach by training groups of rats in three different radial maze paradigms involving forgetting or not (Fig 1). One group of rats was trained in a RM task while two other groups were trained in a WM task involving a simple delayed-non-match-to-place procedure often used to test WM abilities in primates or rodents. One of these two WM groups was trained in a WM task requiring not only the short-term storage of information relevant to an ongoing trial, but also the processing of a high level of proactive interference (PI) caused by repeatedly presenting similar information over a 10-day training period. We previously showed that this high interference WM (HIWM) protocol required the clearance (forgetting) of this interfering information [9]. The second WM group was trained in a low interfering WM (LIWM) protocol so that such forgetting was less required. These three paradigms (RM, HIWM, LIWM tasks) thus tested three conditions gradually involving the adaptive forgetting of previously stored information, with HIWM training being the condition where forgetting of previous trials is the most needed in contrast to the RM task during which such forgetting is deleterious to the consolidation of information into long-term memory. However, to control for motor, motivational and emotional aspects that might be confounding factors to such a comparative approach of different cognitive abilities, we designed these three paradigms so that each day, rats in all conditions visited the same number of arms with the same time interval between each visit. This allows a clear comparison between processes requiring the long-term (RM) or short-term (WM) storage of information and those requiring the adaptive forgetting of previously stored information in WM. To determine such processes, we studied the expression of indirect markers of neuronal activity and synaptic plasticity, *Zif268* and *c-Fos* [22], in the brain of rats trained in one of the three tasks. *Zif268* (also known as *egr-1*, *NGFI-A*, *Krox24* and *Zenk*) and *c-Fos* are two Immediate Early Genes (IEGs) that exhibit a rapid but transient expression upon neuronal activation. *Zif268* has a high level of basal expression in many brain areas as compared to *c-fos* that has a low basal expression level in most neural systems [23, 24]. As biological markers, *Zif268* complements *c-Fos* as it is also largely distributed in many brain regions and, like *c-Fos*, has been linked to learning and memory [25–27]. Using these biomarkers, we showed that the processing of interference in WM might require a specific and negative control of the dentate gyrus (DG) of the dorsal hippocampus materialized by an absence of activation of the expression of both *Zif268* and *c-Fos*. Conversely, we showed that inactivating the DG by ibotenic acid lesion impairs both RM and WM, but seems to improve the processing of interference. The present results thus suggest that the DG is a critical node in processing the forgetting of irrelevant information, an essential process allowing optimal use of cognitive resources.

## Results

### Behavior

The performance of rats trained in the three different tasks (RM, LIWM and HIWM), are represented in Fig 1F. ANOVAs revealed a significant Group effect [F (2, 25) = 108.305; p < 0.0001], a significant Block effect [F (4, 100) = 4.575; p = 0.002], as well as a significant Group x Block interaction [F (8, 100) = 8.157; p < 0.0001]. *Post-hoc* split-by Group analyses revealed that RM rats significantly improved their performance over time [F (9, 36) = 18.413; p < 0.0001] and reached 85% correct choices on the last block of days, indicating a learning of the general rules and strategies required to solve the task. In WM groups, we investigated how proactive interference (PI) affected WM performance. At the beginning of training, both LIWM and HIWM groups started at almost 90% of correct choices. LIWM rats kept high scores throughout the entire experiment, slightly increasing with time and reaching 100% on Block 5 (no significant Block effect). On the contrary, rats trained in the HIWM task showed a decrease in performance over days indicating that accumulation of PI critically distorts WM performance with time. *Post-hoc* Scheffe test run on the performance of rats over the five blocks of days thus revealed a significant difference (p < 0.0001) between the LIWM and HIWM groups, this difference being more salient on the last blocks of days. *Post-hoc* Scheffe tests revealed that a significant difference in score was shown for blocks 4 (p = 0.0033) and 5 (p = 0.0026) respectively between LIWM and HIWM, but not for blocks 1, 2 and 3 (both p > 0.05).

### Immunohistochemistry

**Fig 2:**
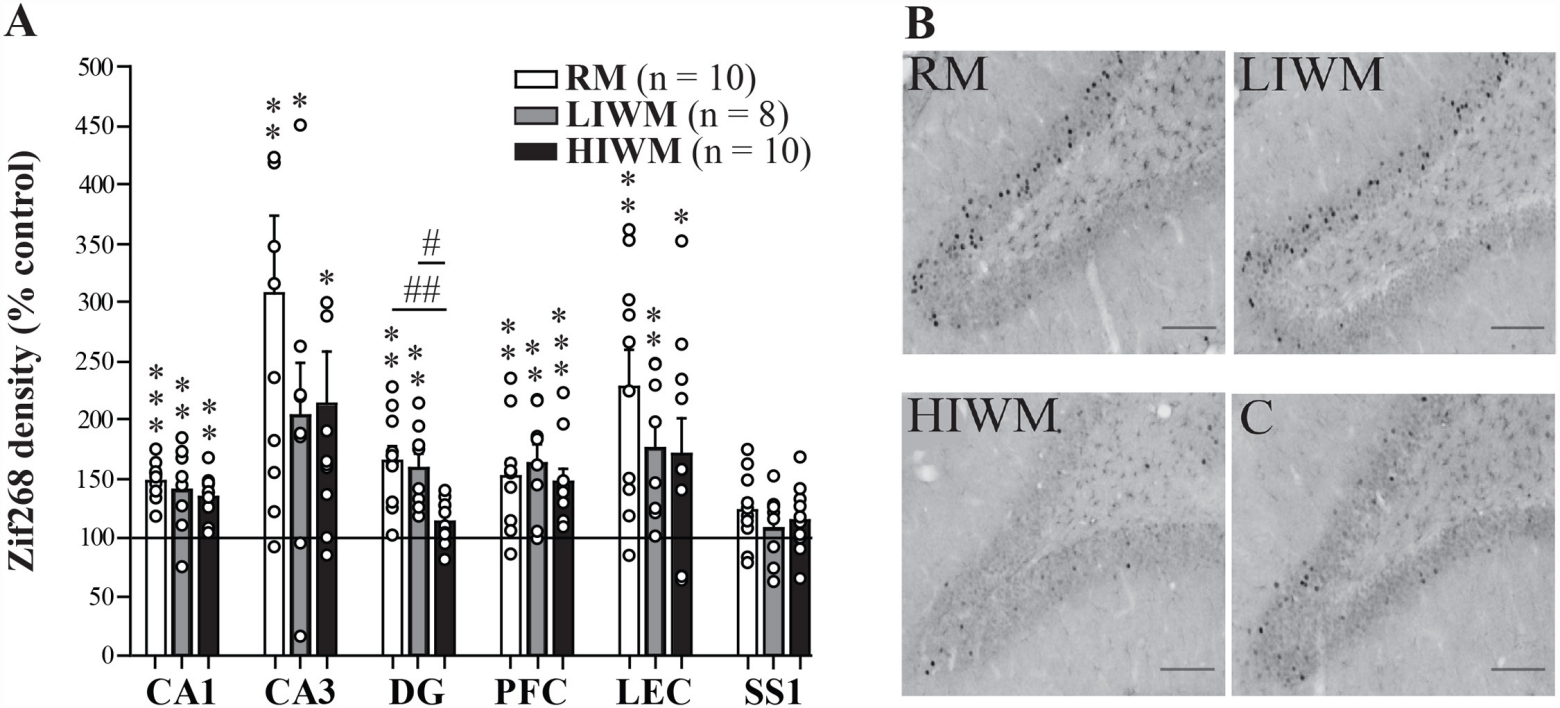
The expression of *Zif268* is decreased in the dentate gyrus of HIWM trained rats as compared to RM or LIWM rats. (A) Density of *Zif268* positive cells in the three experimental groups relative to paired controls (C—black line) in the CA1, CA3 and DG of the dorsal hippocampus, medial prefrontal cortex (PFC), lateral entorhinal cortex (LEC) and primary somatosensory cortex (SS1), after 10 days of training. All groups of rats expressed an increased density of *Zif268* immunoreactive cells in these areas (except the control structure SS1) compared to control animals (n = 16, 100% baseline). This increase was not observed in the DG of HIWM rats (HIWM versus C: Mann–Whitney U-test, p = 0.1876, RM versus C: ** p = 0.0019, ** LIWM versus C: p = 0.004, HIWM versus LIWM: # p = 0.01, RM versus HIWM: ## p = 0.0065). * p < 0.05; **, ## p < 0.01; *** p < 0.001. Dots represent each animal in each group. (B) Representative photomicrographs showing *Zif268*-stained nuclei in the dorsal DG. Scale bar, 100 μm.

**Fig 3:**
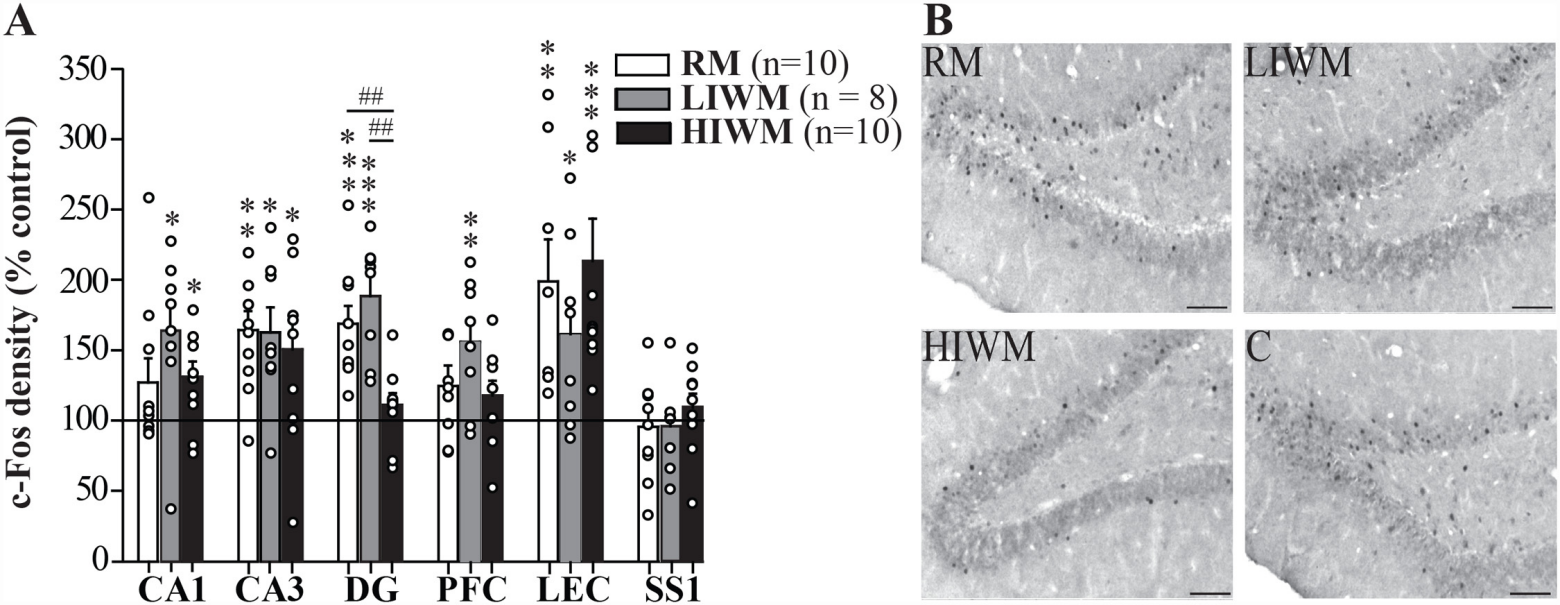
The expression of *c-Fos* is decreased in the dentate gyrus of HIWM trained rats as compared to RM or LIWM rats. (A) Density of *c-Fos* positive cells in the three experimental groups relative to paired controls (black line) in the CA1, CA3 and DG of the hippocampus, medial prefrontal cortex (PFC), lateral entorhinal cortex (LEC) and primary somatosensory cortex (SS1), after 10 days of training. No increase in *c-Fos* expression was observed in the DG of HIWM rats (HIWM versus C: Mann–Whitney U-test, p = 0.3181, RM versus C: *** p = 0.0003, *** LIWM versus C: p = 0.0005, HIWM versus LIWM: ## p = 0.001, RM versus HIWM: ## p = 0.0012). * p < 0.05; **, ## p < 0.01; *** p < 0.001. Dots represent each animal in each group. (B) Representative photomicrographs showing *c-Fos*-stained nuclei in the dorsal DG. Scale bar, 100 μm.

To identify brain regions differentially involved in processing memory over the long term, the short term and the processing of PI, we mapped the regional expression of the IEGs *Zif268* (Fig 2A) and *c-Fos* (Fig 3A) on the last day of training. A significant increase in the density of *Zif268* labeled neurons was observed in the hippocampus (all Mann–Whitney U-tests; CA1: U = 8; p = 0.0001 for RM versus control group (C), U = 21; p = 0.0085 for LIWM versus C and U = 20; p = 0.0016 for HIWM versus C; CA3: U = 22; p = 0.002 for RM versus C, U = 29; p = 0.0321 for LIWM versus C and U = 32; p = 0.0114 for HIWM versus C), lateral entorhinal cortex (U = 18; p = 0.0011 for RM versus control group (C), U = 21; p = 0.0085 for LIWM versus C and U = 43; p = 0.0412 for HIWM versus C) and medial prefrontal cortex (U = 27; p = 0.0052 for RM versus C, U = 18; p = 0.0048 for LIWM versus C and U = 15; p = 0.0006 for HIWM versus C) in all three groups of animals (S1B Fig) compared to a control group (C) composed of rats also exposed to the maze and trained to find food rewards but forced to go into pre-determined arms (see methods and [5]). These results are consistent with the well-established roles of these brain areas in learning and memory [22]. As expected, no such increase was visible in “control regions” such as the primary somatosensory cortex (SS1) (U = 48; p > 0.05 for RM versus control group (C), U = 53; p > 0.05 for LIWM versus C and U = 62; p > 0.05 for HIWM versus C) that was shown not to be specifically activated by higher order cognitive processes [6]. However, we found that the dentate gyrus (DG) of the dorsal hippocampus displayed the most unique pattern of activity, with expression of *Zif268* remaining low after HIWM training (U = 21; p = 0.0019 for RM versus C, U = 17; p = 0.004 for LIWM versus C, U = 55; p = 0.1876 for HIWM versus C, U = 11; p = 0.01 for HIWM vs LIWM and U = 14; p = 0.0065 for RM vs HIWM; Fig 2A and 2B). When mapping the regional expression of *c-Fos*, similar results were found. A significant increase in the density of *c-Fos* labeled neurons was observed in the hippocampus (CA1: U = 56; p = 0.29 for RM versus C, U = 21; p = 0.0118 for LIWM versus C and U = 38; p = 0.0401 for HIWM versus C; CA3: U = 25; p = 0.0055 for RM versus C, U = 22; p = 0.0142 for LIWM versus C and U = 37; p = 0.035 for HIWM versus C), lateral entorhinal cortex (U = 13; p = 0.0047 for RM versus C, U = 24; p = 0.0426 for LIWM versus C and U = 4; p = 0.0002 for HIWM versus C) and medial prefrontal cortex (U = 47; p = 0.2643 for RM versus C, U = 16; p = 0.0091 for LIWM versus C and U = 38; p = 0.094 for HIWM versus C) in all three groups of animals compared to the control group, although this increase was only marginal (and not statistically significant) in CA1 and the prefrontal cortex for the RM group. Similar to *Zif268* expression, no increase in *c-Fos* expression was seen in the SS1 area (U = 74; p > 0.05 for RM versus C, U = 57; p > 0.05 for LIWM versus C and U = 59; p > 0.05 for HIWM versus C), but more importantly, the same pattern of expression was observed in the DG of the dorsal hippocampus, with *c-Fos* expression remaining low after HIWM training (U = 10; p = 0.0003 for RM versus C, U = 6; p = 0.0005 for LIWM versus C, U = 57; p = 0.3181 for HIWM versus C, U = 3; p = 0.001 for HIWM vs LIWM and U = 7; p = 0.0012 for RM vs HIWM; Fig 3A and 3B). Altogether, these results suggest that a non-activation of the DG might be required to accomplish this task and overcome interference.

**Fig 4:**
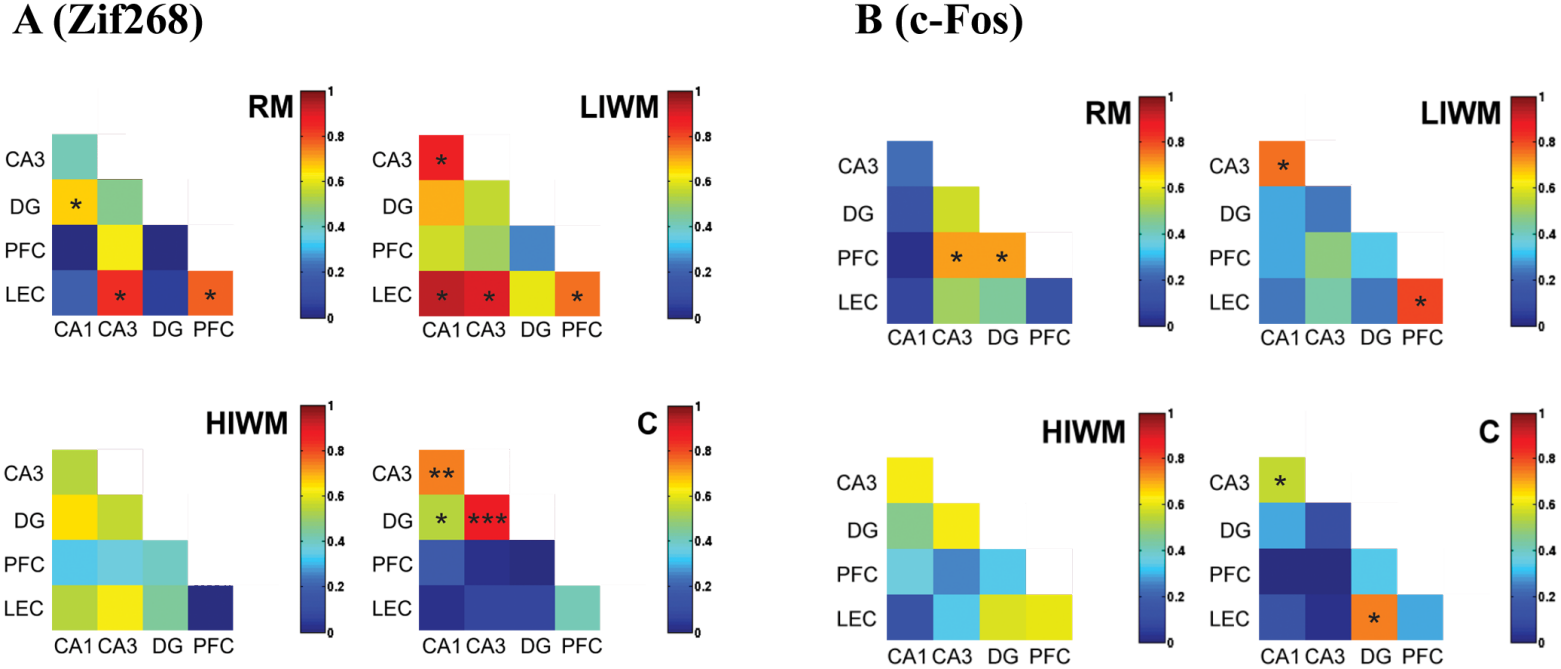
An inter-regional brain de-correlation of functional connectivity is observed after HIWM training. (A) Interregional Correlation matrices for *Zif268* expression within each group. (B) Interregional Correlation matrices for *c-Fos* expression within each group. R-Spearman rank correlation coefficients are color-coded. Colors correspond to values of correlation coefficient *r* (scale, right). Significant correlations (p<0.05) are marked with (*), (p<0.01) are marked with (**) and (p<0.001) are marked with (***).

Using the regional expression of *Zif268* (Fig 4A) and *c-Fos* (Fig 4B–see also S1 Table), we also compared inter-regional brain activity to better understand the functional connectivity existing between brain regions during the different processing of information involved in the three radial maze tasks [6]. In the control group, a high level of positive inter-regional brain correlation was specifically observed between the different areas of the hippocampus (for *Zif268*: between CA1 and CA3 r = 0.73; CA1-DG r = 0.53 and DG-CA3 r = 0.888, p < 0.05; Fig 4A). In addition to the control group, numerous positive correlations were observed between brain regions in the RM (For Zif268: DG-CA1 r = 0.67; LEC-CA3 r = 0.83; LEC-PFC r = 0.769, p < 0.05) and LIWM groups (CA3-CA1 r = 0.86; LEC-CA1 r = 0.93; LEC-CA3 r = 0.905; LEC-PFC r = 0.762, p < 0.05). Although we did not find any correlations existing between IEG expression and behavioral performance (see S2 Table), specific positive correlations are thus evident between intrahippocampal areas but also between the entorhinal and medial prefrontal cortices and the hippocampus. Most interestingly, we found that the pattern of correlation matrix dramatically changed in the HIWM group as compared to the other groups. No inter-regional brain correlation was observed between any of the studied structures (all p > 0.05, except for Zif268 between the DG and the “control” region SS1: r = 0.745; p = 0.013) suggesting that forgetting and the processing of PI may require de-coupling within these memory circuits. Together with a non-activation of the DG, this inter-regional brain de-correlation might specifically promote forgetting of previous trials required to perform the HIWM task.

### Lesion study

**Fig 5:**
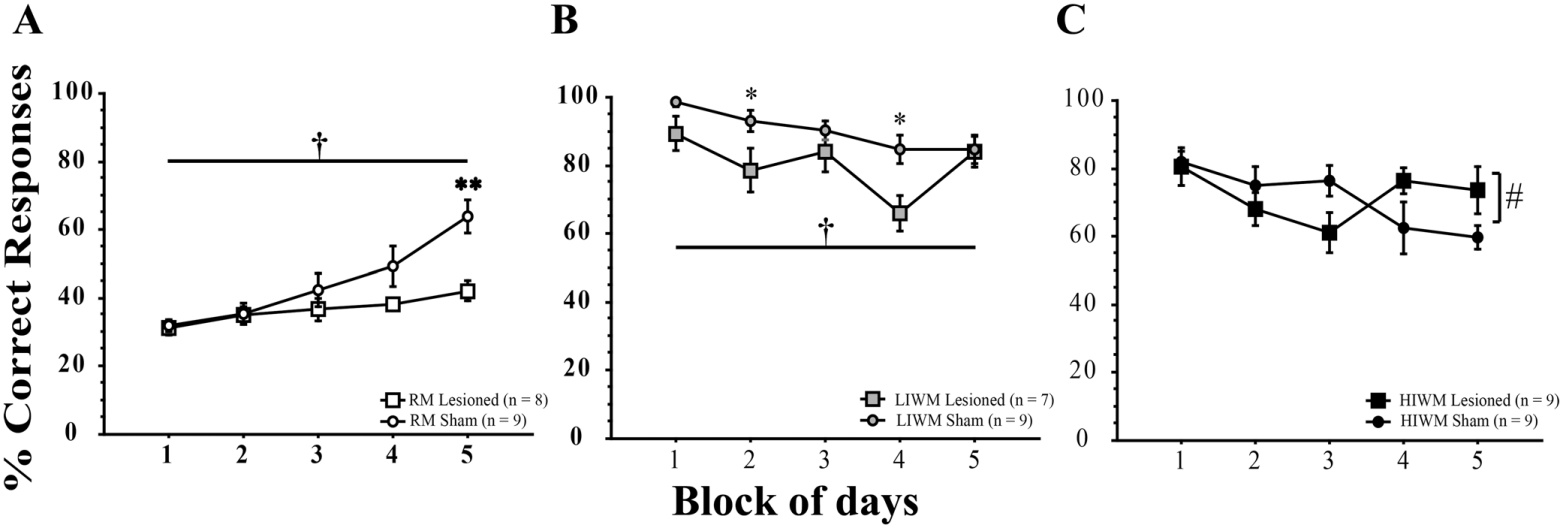
Ibotenic acid lesion of the DG impairs RM and WM but improves the processing of interference. (A-C) Percentage of correct choices ± s.e.m per Block of days in RM (A), LIWM (B) and in HIWM (C) for ibotenic acid lesioned and control animals. † Group effect; * Block effect; * p < 0.05; ** p < 0.01; # interaction p < 0.05.

Our findings that the dorsal DG shows no increase in *Zif268* or *c-Fos* expression after radial maze training involving forgetting are particularly striking. To what extent is the DG required for WM and the processing of PI? To address this question, we examined the effects of inactivating the DG by ibotenic acid lesion on performance of the three behavioral tasks we already described. We first verified that the lesion was restricted to the DG. While the CA1 and CA3 subfields of the dorsal hippocampus were spared by the ibotenic acid injection in the DG, granule cells of the DG were almost completely eliminated compared to sham-operated rats (S2A and S2B Fig). A part of CA3 comprised in the *hilus* was however also lesioned by our procedure. Fig 5A shows the acquisition curve of the rats trained in the RM task. In this task, DG lesioned animals exhibited a marked impairment in performance as compared to the Sham-operated group. ANOVAs revealed a significant Group effect [F (1, 15) = 4.89; p = 0.0429], a significant Block effect [F (4, 60) = 16.10; p < 0.0001], as well as a significant Group x Block interaction [F (4, 60) = 4.61; p = 0.0026]. *Post hoc* split-by Group analyses revealed that sham-operated rats significantly improved their performance over time (significant Block effect; [F (4, 32) = 15.351; p < 0.0001]) unlike DG lesioned rats (marginal Block effect; [F (4, 28) = 2.732; p = 0.0489]). Similarly, DG lesioned rats showed a net impairment when trained in the LIWM task (Fig 5B) as compared to sham-operated rats. ANOVAs revealed a significant Group effect † [F (1, 14) = 10.419; p = 0.0061] and a significant Block effect * [F (4, 56) = 4.535; p = 0.0030] (on Block 2 and Block 4, LIWM lesioned versus LIWM Sham; p < 0.05). *Post hoc* split-by Group analyses revealed that LIWM lesioned rats exhibited impaired performances over time (p = 0.0438). In sharp contrast, the lesion of the DG did not seem to impair performance when rats were trained in the HIWM protocol (Fig 5C). In fact, after a small but non significant drop observed in Block 3, DG lesioned HIWM rats performance seem to be enhanced as compared to sham-operated rats at the end of training. ANOVAs revealed a significant Group x Block interaction [F (4, 64) = 2.784; p = 0.0339]. *Post hoc* split-by Group analysis revealed that sham-operated rats exhibited impaired performances over time due to the build-up of interference (significant Block effect [F (4, 32) = 3.569; p = 0.0162]) as observed in intact animals in experiment 1. In contrast, DG lesioned rats were immune to interference and did not exhibit impairment in performance (no significant Block effect, [F (4, 32) = 1.671; p = 0.1810]). This result is in agreement with our IEG data suggesting that a non-activation of the DG is required for the processing of interference.

## Discussion

Altogether, the results presented here confirm previous studies showing that the hippocampus is involved and required for RM and WM storage [5, 6, 28, 29]. However, we show for the first time that within the hippocampus, the processing of PI and forgetting of previously stored, but non-relevant information results in a selective absence of activation of the DG. We also show that inactivating this structure (by lesion) does not impair, but facilitate the processing of PI suggesting that the inhibition of the DG could be required to process and forget non-useful information.

The behavioral results of our first experiment (Fig 1F) show that rats could effectively learn the RM task we designed. RM rats thus showed a significant increase in performance over the 10-day training period. In contrast, all WM rats displayed a high percentage of correct responses from the first Blocks of training. This immediate learning of the WM task delayed-non-match rule is certainly due to innate spontaneous alternation, a behavior that naturally causes rodents to choose a different option (visit arm#2) than one previously adopted (visit arm#1) and in consequence to alternate exploration between two open arms [30]. This tendency to spontaneously alternate between radial maze arms facilitates correct non-match responses. Nevertheless, spontaneous alternation requires memory storage of the previously visited arm in order to alternate to a different arm, and has long been shown to be dependent on the hippocampal formation functional integrity [31]. Most interestingly, while LIWM rats kept high scores throughout the entire experiment, HIWM rats showed a significant decrease in their performance over the course of training. This significant decrease is attributed to the high level of interference and repetition present in the HIWM task. This modification in WM performance due to the ever-increasing buildup of PI has already been observed in our previous experiments using similar WM paradigms [10, 32]. This result highlights an often overlooked issue concerning WM; more precisely, that information supposedly stored temporarily in WM can have an impact on the long-term when it becomes an interference for subsequent WM information as seen here with the decrease in performance over days (not seconds or minutes) in the HIWM group (see Fig 1F). Information supposedly stored in short-term/WM thus outlast their purpose by interfering, several days later, with the storage of newer information. In consequence, this result questions the existence of a pure short-term memory store [33, 34] and rose doubts about a dissociation between short-term and long-term memory. Ranganath and Blumenfeld (2005) thus argued that the evidence suggesting distinct neuroanatomical substrates for short and long-term memory may have been misleading, and reviewed evidence demonstrating that short-term memory might be simply considered a temporary activation of some information already stored in long-term memory [34]. Various studies have shown similarities in the neural correlates of long-term memory and WM suggesting that these different cognitive functions activate overlapping brain regions [35]. Our data seem to confirm such findings as both RM, LIWM and HIWM training induces a similar pattern of IEGs expression, with the exception of the DG that was found to be non-activated by HIWM.

*Zif268* expression in pyramidal cells of the CA1 and CA3 areas of the dorsal hippocampus, the entorhinal cortex and the prefrontal cortex were significantly elevated after RM and WM training, and this elevation was not altered by the presence of PI. Slight difference with the pattern of expression of *c-Fos* in these structures could be observed. RM thus failed to increase expression of *c-Fos* in CA1 or the prefrontal cortex. Differences in the expression of *Zif268* and *c-Fos* have been previously reported and may explain such discrepancies [5, 26]. In rats, we have thus repeatedly observed that CA1 expression of *c-Fos* is much weaker compared to the one of *Zif268* (PA Salin and MA Joseph’s personal observations). Such weak expression of *c-Fos* in CA1 may thus account for a failure to show a significant increase in *c-Fos* expression in this area for RM rats. However, overall, the pattern of expression of *c-Fos* largely reflects the one of *Zif268*. These results suggest that, like RM, WM depends on the activation of the hippocampal complex and of the prefrontal cortex. These data are consistent with previous studies that found an implication of the dorsal hippocampus as well as the prefrontal cortex in WM [36]. In contrast, our data revealed a potential contribution of the DG of the dorsal hippocampus in processing PI. While a classic WM task (LIWM) and a RM task increased the activation of IEGs in the DG, a WM task involving the processing of PI (HIWM task) caused a non-activation of this structure. In addition, such a task also causes de-coupled functional connectivity as no inter-regional brain correlation was observed between any of the studied structures. This result suggests that forgetting and the processing of PI may both require an inactivation of the DG and a functional de-coupling within the memory circuits. We thus assessed the effect of a specific inactivation of the DG on behavioral performance of rats tested in our three different radial maze paradigms, and found that a lesion of the DG impairs RM and LIWM training, but in contrast seems to improve the processing of interference in the HIWM task, at the end of the learning curve, when the gradual build-up of PI was observed in non lesioned rats. This facilitation may occur because lesion of the DG prevents the recall of similar but irrelevant information previously stored in memory from interfering with the recall of newer information. This result confirms previous data from our team showing that RM and WM (requiring the processing of interference) are somewhat antagonistic processes [9] as DG lesion impairs the storage of information but benefits WM by facilitating the processing of interference. In addition, confirming our immunohistochemical findings, these data also seem to demonstrate that an inhibition of the DG is required to overcome and forget interfering non-relevant information.

The DG has largely been shown to be involved in pattern separation [37–39], a process by which the amount of overlap between two representations stored in memory can be reduced. By using electrophysiology and functional anatomy, it has been shown that the population of activated neurons is different when rats are placed in slightly different environment [40]. Thus, the function of pattern separation is to make different, but quite similar representations more distinct in order to afford rapid learning without inducing interference and retrieval errors [41]. Shutting down this DG-dependent pattern separation function may be necessary for the subject to focus on an ongoing trial, especially in task involving a high level of overlap between different trials (HIWM task). By reducing the number of active cells in the DG, the animal may thus be able to ignore and forget previous similar (but irrelevant) representation/trial stored in memory (e.g. trials of days 1 to 7, see Fig 1) and thus perform correctly an ongoing trial (e.g. during day 8). Our results suggest that processing interference in a WM task could specifically require an inhibition of the DG, a site where adult neurogenesis is known to occur [37, 38]. They are thus in agreement with work from our group showing that DG inhibition of neurogenesis improves WM performance, especially in tasks where repetitive information were presented as it is the case in a HIWM task [32, 42]. Future experiments are required to establish if the number of activated DG newborn neurons decreases selectively in the HIWM task. Given the importance of forgetting of irrelevant information for optimal use of memory in everyday life, it is now crucial to understand the molecular and cellular mechanisms underlying this essential cognitive function. Much work still needs to be done to achieve this goal, but the results presented in this study provide new insights in the molecular bases of adaptive forgetting by asserting the DG as a critical node in this process.

## Material and Methods

### Subjects

A total of 95 three months old Dark Agouti rats initially weighing 200-250g at the beginning of the experiment were purchased from Harlan France. They were kept in a 12/12h light/dark cycle with *ad libitum* access to food and water but were subsequently food deprived and maintained at 85% of their free-feeding weight throughout the experiment. For the behavioral and immunohistochemical experiment, rats (n = 44) were housed in cohorts of two. Three groups learned a radial maze task (High Interference Working Memory HIWM, Low Interference Working Memory LIWM and Reference Memory RM group). Three groups served as controls (Yoked HIWM or YHIWM, Yoked LIWM or YLIWM, and Yoked RM or YRM). For the behavioral lesion study, the animals (n = 52) were also housed in pairs so that a lesioned rat could be housed with a sham-operated control. Six groups of rats thus learned a task (DG lesioned RM, LIWM, and HIWM rats and their respective controls sham-operated RM, LIWM, and HIWM rats). One animal from this lesion group died during surgery. The final number of animal for this lesion study was n = 51. This study was carried out in strict accordance with the recommendations of the Lyon1 University ethical committee for the use of experimental animals and of the European committee (2010/63/EU). The protocol was approved by the Lyon1 University ethical committee for the use of experimental animals (Permit Number: CE2A-UCBL 55). All efforts were made to minimize suffering.

### Behavioral Apparatus

An eight-arm radial maze [3] requiring the use of spatial orientation and memory was used for both experiment 1 and 2. The apparatus consisted of an elevated radial maze from which eight arms (87 cm long x 12 cm wide) radiated from an octagonal central platform (34 cm diameter). The entrance of each arm was blocked by opaque Perspex doors that could be automatically lowered (pneumatic system) by the experimenter located in a room directly adjacent to the testing room. Squared food wells of 2cm diameter and 0.5cm deep were fixed at 0.5 cm at the end of each arm. Food rewards (Dustless Precision Pellets; Bioserve, Frenchtown, NJ) could be placed in these food wells (Fig 1D). The maze was located in a room with a number of extra-maze cues (e.g., poster, door, furniture). A video camera, connected to a video recorder and a monitor, was fixed above the maze to record the animal’s behavior.

### Behavioral protocol

Food deprived rats had to retrieve food rewards (sugar pellets, Bioserv) at the end of the maze’s arms using spatial navigation and distal visual cues surrounding the maze. Rats underwent a 6-day habituation period during which they became accustomed to the radial maze environment and learned to find rewards in the arm wells. After habituation, they were pseudo-randomly assigned to one of the following groups (see below); whatever their group assignment, they were able to complete eight runs to an arm per day, making the three tasks strictly comparable in terms of motivational, emotional and motor processes:

#### Reference Memory group

Rats trained in the RM task had to retrieve food pellets in two arms of the maze (Fig 1C). These two arms remained constant and were the same every day for the entire 10 days (= 5 Blocks of 2 daily sessions) of training [28]. Rats were initially placed in a pseudo-randomly chosen starting arm at which point all arms of the maze were opened. A transparent blocker prevented rats from going to the food well of the starting arm. Once a rat chose one of the arms (an arm selection was defined when the animal reached the arm’s half way) the door to that arm was closed, confining the rat in the chosen arm. After consuming the food reward in the case of a correct choice, or not in the case of an incorrect choice, rats were returned to a transfer cage adjacent to the maze for a short delay of 15 seconds. The doors to previously chosen arms remained closed until both food rewards were retrieved in order to prevent the rat to return into such arms (committing WM errors). Once the two food pellets were retrieved, the two previously baited arms were re-baited and all arms were re-opened. Rats underwent eight trials per day (one trial = one run into an arm) and the maximum score per day was fixed at 8 pellets eaten. The latency to choose an arm as well as the number of correct choices were scored. Half of the experimental RM rats were paired with yoked control (YRM) rats that performed the same amount of motor activity and ate the same number of pellets. These yoked controls were forced to enter into pseudo-randomly chosen arms and were either reinforced or not depending on the performance of their experimental matched rat. The starting and destination arms varied between trials in such a way that yoked controls could not use motor memory to predict which direction they had to go or the outcome of a given run (reinforced or not). The use of yoked controls allows the experimenter to conclude that all differences seen between groups after immunohistochemistry analysis are inherent to learning processes during the task and not due to motivational, sensory or locomotor aspects of the task [5].

#### Low interference Working Memory (LIWM) group

The WM tasks consisted in a delayed-non-match-to-place task classically used in various models ranging from rodents to humans to assess WM. Rats trained in the LIWM task were submitted to four trials per day, each consisting of a sample and a choice phase (matching the eight runs performed by the RM group–see Fig 1D). In the sample phase, rats were first allowed, from a starting arm, to enter one randomly chosen baited arm while all other arms remained closed. Rats were then returned to the transfer cage for a short delay of 15 seconds (identical delay than in the RM task). During the subsequent choice phase, rats were presented with two adjacent arms: the arm that had just been visited and empty of food, and a new adjacent arm containing a second food reward. Rats had to choose the novel arm in order to be positively reinforced (classical delayed non-match to place task). Different pairs of arms were used for each trial (Fig 1D indicates an example of trial sequence for a given day), and the sample presentation of the first baited arm (right or left arm of the pair) was pseudo-randomly determined. As described above for RM rats, half of the experimental LIWM rats were paired with yoked controls (YLIWM) that were exposed to the same radial maze arms. The two groups (LIWM and YLIWM) were thus exposed to the same spatial information. Whereas LIWM rats had to learn a delayed non-match to place task rule in order to successfully complete the task, YLIWM rats were exposed to an equal number of non-match and match runs in a pseudorandom fashion in order to prevent YLIWM rats to predict the outcome of a trial. Like YRM rats, YLIWM rats were forced to visit only one arm during each run and were not exposed to any cognitive choice as compared to LIWM rats.

#### High interference Working Memory (HIWM) group

HIWM rats were exposed to an experimental procedure similar to the one used in the LIWM task, except that the same pair of arms was used everyday for each trial. We have previously shown that this task promoted high level of interference, and requires the necessity to forget previous trials in order to correctly complete an ongoing trial [9, 17, 32] (Fig 1E indicates an example of trial sequence for a given day). Half of the experimental HIWM rats were paired with yoked controls (YHIWM) that performed the same amount of motor activity and ate the same number of pellets as already described for the YRM and YLIWM groups.

### Immunohistochemistry

Ninety minutes after the last training session (time required to induce expression of *Zif268* and *c-Fos* [22]), rats (n = 44) were deeply anesthetized with an overdose of sodium pentobarbital (140 mg/kg, Sigma) and then transcardially perfused with 100 ml heparinized ringer lactate, followed by 4% paraformaldehyde in 0.1 M phosphate buffer (pH 7.4). Brains were then removed from the skull and were cryoprotected in 30% phosphate buffered sucrose. Brains were cut (25 μm thin sections) on a freezing cryostat. Serial sections were collected in PBST Azide and then incubated at 4°C for 6 days with rabbit polyclonal antibody for *Zif268* (1:1000, Santa Cruz Biotech) or 3 days with rabbit polyclonal antibody for *c-Fos* (1:5000, Calbiochem). Sections were incubated with a biotinylated secondary antibody IgG (donkey-anti-rabbit, 1:1000, Rockland, Tebu-bio) overnight at 4°C. The next day, sections were processed with avidin-biotin horseradish peroxidase complex (ABC 1:2000, Elite Kit from Vector Laboratories). Finally, immunoreactivity was visualized with 0.025% diaminobenzidine chromogen (DAB, Sigma), 0.05% Nickel and 0.03% H2O2 as reaction initiator. Sections were mounted on gelatin-coated slides, dehydrated through a graded series of alcohols and coverslipped.

### Cell counts

Quantitative analyses of *Zif268* and *c-Fos* positive cells were performed by using a computerized image processing system (Mercator, Explora Nova ^®^) coupled to an optical upright microscope. Structures were defined according to the Paxinos and Watson atlas [43]. Immunoreactive neurons were counted bilaterally using a minimum of four sections. Cells were counted throughout the different area of the sections with an objective of 20x magnification. Data from YLIWM, YHIWM and YRM were pooled (control group—C) as no significant statistical difference was found between these groups in either *Zif268* or *c-Fos* activation in all studied structures. For each animal, the density of *Zif268* and *c-Fos* positive cells was calculated by dividing cell counts of each area by the surface of the area. Each animal’s areas density was then normalized by dividing the corresponding control density (% of control).

### Surgery–Dentate Gyrus lesion

As the result of the immunohistochemical study revealed that the DG may play a differential role in memory and forgetting, we conducted a lesion experiment. 51 rats were thus allocated to either the bilateral DG lesion group (n = 24) or the sham operated group (n = 27). Surgery was performed under Isoflurane anesthesia in a standard stereotaxic apparatus. The rats were pre-anesthetized in a rectangular (30x20x15cm) chamber in order for them to endure positioning on the stereotaxic frame. Anesthesia was maintained via an inhalation nose cone affixed to the mouth bar on the frame. Inspired concentrations of 2–3% Isoflurane with oxygen are required for the induction and later on around 1.5% for the maintenance of the narcosis. As preparation for surgery, ophthalmic liquid gel was applied to the rat’s eyes for protection, the hair was shaved from the top of the rat’s head with an electric shaver and the scalp was cleaned with Betadine. A 2 cm midline incision was made and the skull disclosed. The skin was retracted with 4 Bulldog clamps to expose the skull and hold opened the incision. Holes were drilled into the skull bilaterally over the DG. The dura was removed using a small syringe. For the lesion animals, 4 holes were drilled bilaterally over the DG using coordinates derived from pilot experiments. The lesion coordinates for the dorsal DG group are 2.7 mm posterior to bregma, 1.2 mm lateral to midline, and 3.4 mm ventral from skull, and 3.5 mm posterior to bregma, 1.9 mm lateral to midline, and 3.0 mm ventral from skull. Glass tubing with microcapillary (Harvard apparatus) was used for iontophoresis ejections. They were pulled in a single step on a Sutter PE-2 vertical puller (Narishige, Japan). The heat was programmed to give a tip of a less than 1 μm diameter size. Then, this tip was broken at 5 μm diameter to allow ibotenic acid ejection. Ibotenic acid (Tocris, bioscience) dissolved in sodium chloride to 10 mg/ml (pH = 8) was ejected at 4 sites in each hemisphere. The pipette was left in place for 5 min before ejection. For iontophoresis, the pipette was connected by a silver wire immersed in the ibotenic acid solution to a current generator (CS4, Transkinetics, MA) that delivered pulsed negative current (7 seconds on/7 seconds off) for 4 minutes. At each site, ibotenic acid or NaCl were administered iontophoretically using currents of -12 μA. At the end of ejection, the pipette was left in place for at least 5 minutes to avoid leakage of the ibotenic acid along the pipette track. After surgery, the skin was sutured and local antiseptic was applied to prevent post-surgery infection. 2 ml of a 5g/l glucose solution was injected subcutaneously. The animals were then allowed a post-surgical recovery period of 10 days before starting behavioral testing in the radial maze as described above.

### Statistical analysis

#### Behavior (immunohistochemical study)

Behavioral data were analyzed using two-way ANOVAs (Analysis of Variance) for repeated measures with Block (2 days) and Group (RM, LIWM, HIWM) as main factors (Statview 5.0.). Further comparisons were performed by *post hoc* (Scheffe and split-by Group) analyses for particular within-group comparisons. Data are expressed as means ± s.e.m.

#### Immunohistochemistry

*Zif268* and *c-Fos* immunoreactivity was statistically analyzed with Mann-Whitney U-test. Data are expressed as mean of normalized *Zif268/c-Fos* density (% of control) ± s.e.m.

#### Correlation

The density of *Zif268* and *c-Fos* labeled neurons was also used to compare inter-regional brain activity and to better understand the functional connectivity existing between brain regions during the different cognitive processes studied here [6]. A correlation matrix was thus constructed based on this *Zif268* and *c-Fos* density for each experimental group using the Spearman’s rank correlation coefficient, a measure of statistical dependencies between non-parametric variables. A positive correlation coefficient between two brain regions indicates that an increase in a region results in a proportional increase in the other region.

#### Dentate Gyrus lesion

Behavioral data from the lesion study were analyzed using two-way ANOVAs (Analysis of Variance) for repeated measures with Block (2 days) and Group (RM lesioned versus sham; LIWM lesioned versus sham; HIWM lesioned versus sham) as main factors (Statview 5.0.). Further comparisons were performed by *post hoc* (Scheffe and split-by Group) analyses for particular within-group comparisons. Data are expressed as means ± s.e.m. Subsequent histological analyses revealed that lesions were limited to the dorsal DG with negligible damages in other areas (S2 Fig).

## Supporting Information

S1 Fig (A) Diagrams of rat brain coronal sections depicting regions of interest (filled areas) where immediate-early gene cell counts were obtained. The numbers indicate the distance (in millimeters) of the sections from bregma [43]. aCC: anterior cingulate cortex; dCA1: CA1 field of dorsal hippocampus; dCA3: CA3 field of dorsal hippocampus; DG: dentate gyrus; IL: infralimbic cortex; LEC: lateral entorhinal cortex; PrL: prelimbic cortex; SS1: primary somatosensory cortex; IEG counts for the following brain regions were expressed as the pooled means of the listed subregions: Prefrontal cortex: IL, PrL, aCC. (B) Representative Photomicrographs from each region of interest showing *Zif268*-stained nuclei in the dorsal CA1, CA3 and dentate gyrus of the hippocampus, prefrontal cortex and somatosensory cortex in our four groups of rats. Scale bar, 100 μm
(TIF)

S2 Fig (A) Illustration showing the extent of the lesions to the Dentate Gyrus. The largest and the smallest tissue damage produced by ibotenic acid in the dorsal hippocampus are shown in gray and black respectively. The numbers represent distance (mm) from bregma. (B) Photomicrograph of Dentate Gyrus in a lesioned animal (right) and a sham animal (left) stained with *NeuN*. In this example, infusions of ibotenic acid produced a loss of tissue of the dentate gyrus. Scale bar, 150 μm. Atlas sections are from the Paxinos and Watson [43].
(TIF)

S1 Table Interregional Correlation matrices for *Zif268* (A) and *c-Fos* (B) expression within each group. R-Spearman rank correlation coefficients are indicated in the tables. Correlation coefficients are in bold when significant (p<0.05).
(PDF)

S2 Table Correlation matrices between Zif268 (left) and c-Fos (right) expression within each group and performance on the last day of training. R-Spearman rank correlation coefficients are indicated in the tables. No significant correlation was found. For LIWM, such correlation could not be computed as all animals for this group displayed a score close or equal to 100% at the end of training.
(TIF)
