# Supplementary figures and images for: Differential Involvement of the Dentate Gyrus in Adaptive Forgetting in the Rat
Source: PLoS One. 2015 Nov 3;10(11):e0142065. doi: 10.1371/journal.pone.0142065 (PMC4631520; doi:10.1371/journal.pone.0142065)

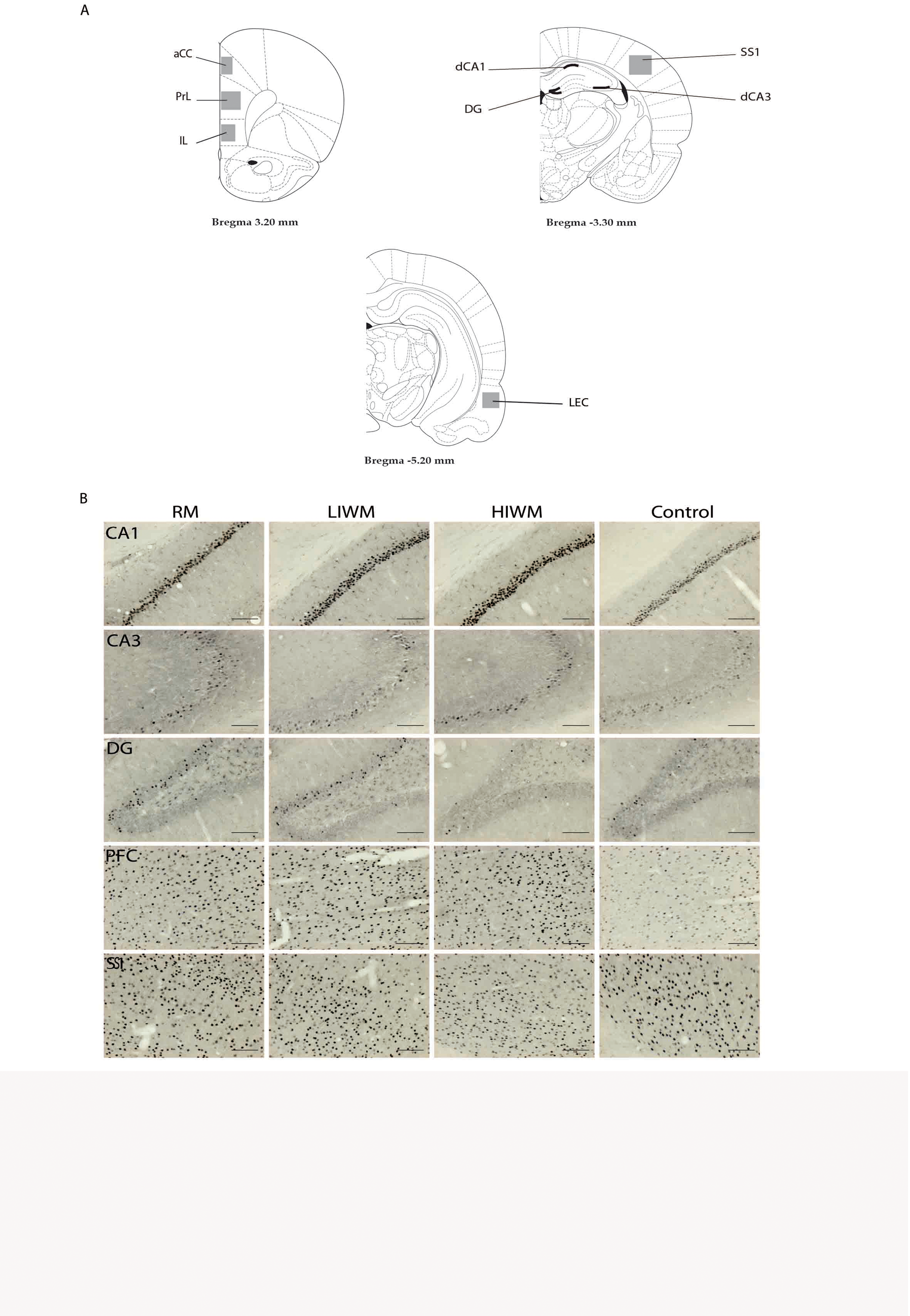

Supplement: S1 Fig — (A) Diagrams of rat brain coronal sections depicting regions of interest (filled areas) where immediate-early gene cell counts were obtained. The numbers indicate the distance (in millimeters) of the sections from bregma [43]. aCC: anterior cingulate cortex; dCA1: CA1 field of dorsal hippocampus; dCA3: CA3 field of dorsal hippocampus; DG: dentate gyrus; IL: infralimbic cortex; LEC: lateral entorhinal cortex; PrL: prelimbic cortex; SS1: primary somatosensory cortex; IEG counts for the following brain regions were expressed as the pooled means of the listed subregions: Prefrontal cortex: IL, PrL, aCC. (B) Representative Photomicrographs from each region of interest showing Zif268-stained nuclei in the dorsal CA1, CA3 and dentate gyrus of the hippocampus, prefrontal cortex and somatosensory cortex in our four groups of rats. Scale bar, 100 μm (TIF) [file pone.0142065.s001.tif]

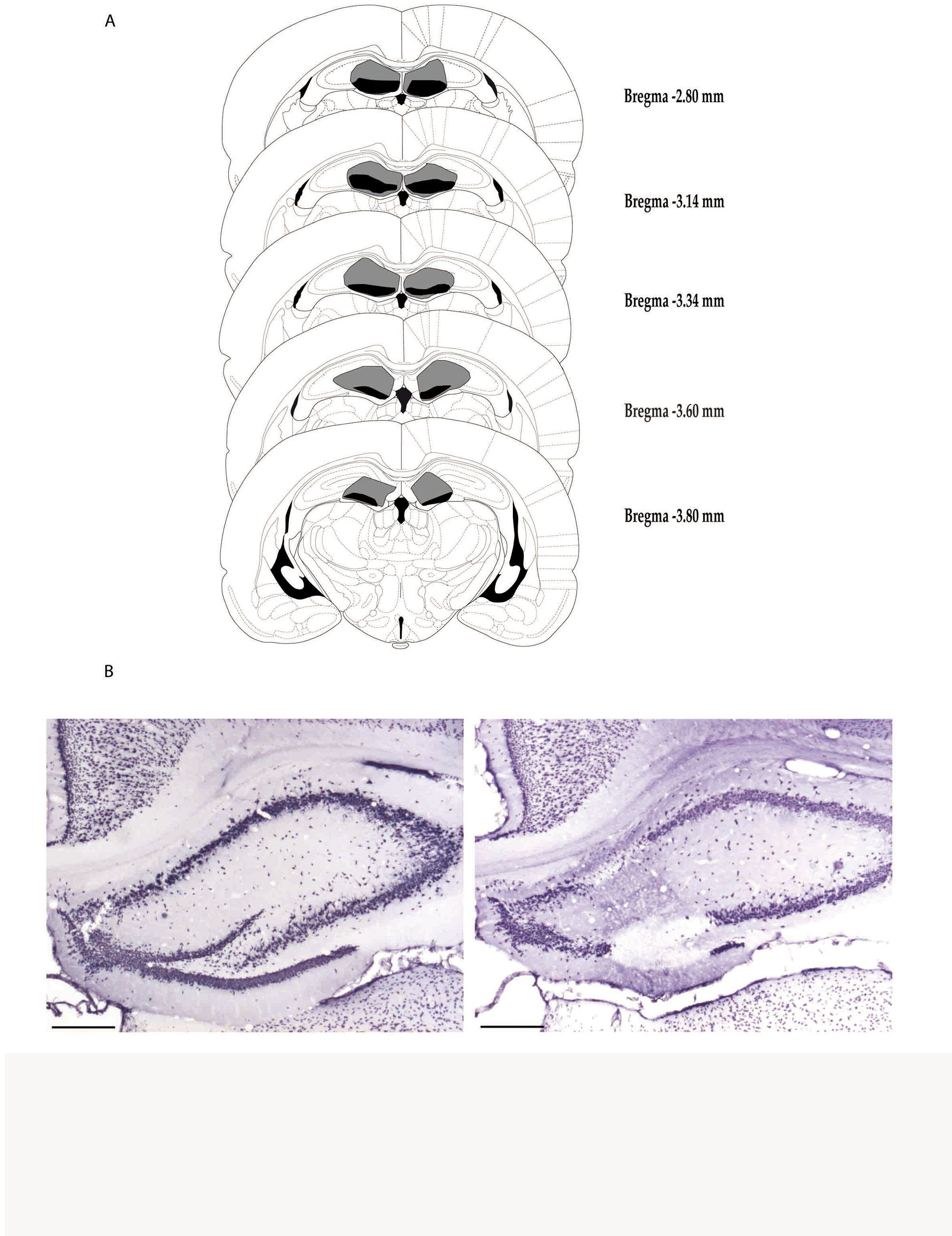

Supplement: S2 Fig — (A) Illustration showing the extent of the lesions to the Dentate Gyrus. The largest and the smallest tissue damage produced by ibotenic acid in the dorsal hippocampus are shown in gray and black respectively. The numbers represent distance (mm) from bregma. (B) Photomicrograph of Dentate Gyrus in a lesioned animal (right) and a sham animal (left) stained with NeuN. In this example, infusions of ibotenic acid produced a loss of tissue of the dentate gyrus. Scale bar, 150 μm. Atlas sections are from the Paxinos and Watson [43]. (TIF) [file pone.0142065.s002.tif]

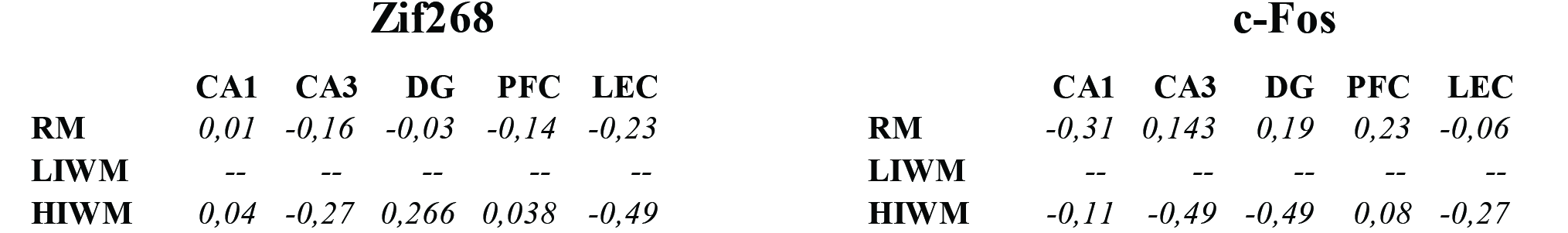

Supplement: S2 Table — R-Spearman rank correlation coefficients are indicated in the tables. No significant correlation was found. For LIWM, such correlation could not be computed as all animals for this group displayed a score close or equal to 100% at the end of training. (TIF) [file pone.0142065.s004.tif]
