# Supplementary material for: Differential Involvement of the Dentate Gyrus in Adaptive Forgetting in the Rat
Source: PLoS One. 2015 Nov 3;10(11):e0142065. doi: 10.1371/journal.pone.0142065 (PMC4631520; doi:10.1371/journal.pone.0142065)
Supplement: S1 Table — R-Spearman rank correlation coefficients are indicated in the tables. Correlation coefficients are in bold when significant (p<0.05). (PDF) [file pone.0142065.s003.pdf]

A

Zif268

RM	CA1	CA3	DG	PFC	LEC
CA1	1				
CA3	0,42	1			
DG	**0,67**	0,467	1		
PFC	-0,1	0,612	0,006	1	
LEC	0,2	**0,83**	0,054	**0,769**	1

LIWM	CA1	CA3	DG	PFC	LEC
CA1	1				
CA3	**0,86**	1			
DG	0,69	0,547	1		
PFC	0,57	0,5	0,262	1	
LEC	**0,93**	**0,905**	0,595	**0,762**	1

HIWM	CA1	CA3	DG	PFC	LEC
CA1	1				
CA3	0,53	1			
DG	0,64	0,552	1		
PFC	0,33	0,358	0,394	1	
LEC	0,54	0,612	0,442	-0,01	1

Control	CA1	CA3	DG	PFC	LEC
CA1	1				
CA3	**0,73**	1			
DG	**0,53**	**0,888**	1		
PFC	0,17	-0,12	-0,02	1	
LEC	-0,02	0,059	0,047	0,4	1

B

c-Fos

	CA1	CA3	DG	PFC	LEC
CA1	1				
CA3	0,224	1			
DG	0,127	0,576	1		
PFC	-0,03	**0,709**	**0,71**	1	
LEC	0,071	0,5	0,45	0,12	1

	CA1	CA3	DG	PFC	LEC
CA1	1				
CA3	**0,762**	1			
DG	-0,29	0,238	1		
PFC	0,286	0,476	0,33	1	
LEC	0,238	0,428	0,24	**0,81**	1

	CA1	CA3	DG	PFC	LEC
CA1	1				
CA3	0,624	1			
DG	0,454	0,624	1		
PFC	0,366	0,25	0,33	1	
LEC	0,115	0,333	0,59	0,6	1

	CA1	CA3	DG	PFC	LEC
CA1	1				
CA3	**0,553**	1			
DG	0,286	0,107	1		
PFC	-0,56	-0,57	0,34	1	
LEC	0,126	0,016	**0,74**	0,29	1
